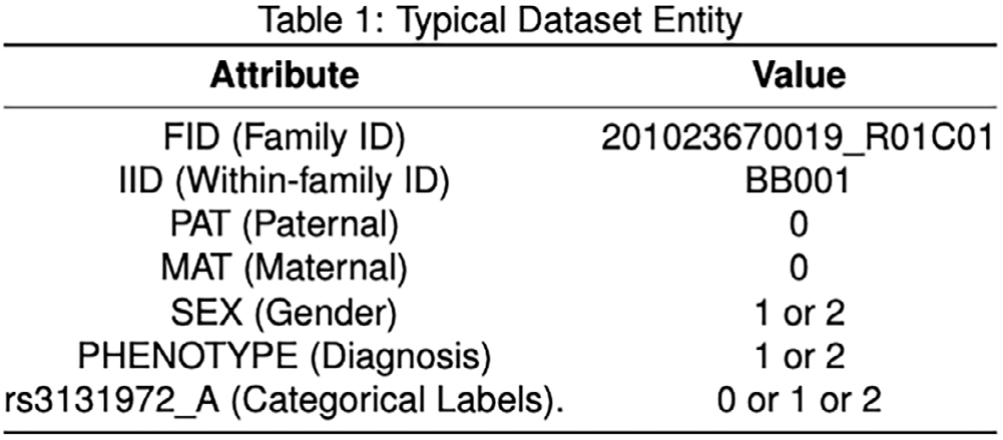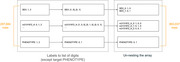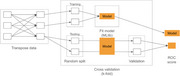# A Data‐driven Approach for Genetic Prediction of Alzheimer's Diseases when Polygenic RiskScores Fail to account AD's Genetic Complexity

**DOI:** 10.1002/alz70855_106728

**Published:** 2025-12-24

**Authors:** Aditya Purswani, Thomas Goddard, Keeley J Brookes, Anto Praveen Rajkumar Rajamani, Parth Patel, Anasuya Dutta, Weiyi Sun, Armaghan Moemeni

**Affiliations:** ^1^ University of Nottingham, Nottingham, Nottinghamshire, United Kingdom; ^2^ Nottingham Trent, Nottingham, United Kingdom

## Abstract

**Background:**

Alzheimer's Disease (AD) is the primary cause of cognitive decline in individuals. Early diagnosis of AD is significantly difficult because of its complex pathogenesis and overlap with symptoms of aging, yet crucial for early treatment. Genetic phenotyping addresses this issue by identifying at‐risk individuals. Polygenic Risk Scores (PRS) are common genetic prediction tools but they don't capture AD's genetic complexity. This study uses advanced AI and Data Science techniques to enhance the predictive accuracy of Alzheimer's and reduce computational time by using advanced global AI approaches.

**Method:**

The dataset contains 534 individuals (AD and healthy controls) from the Brains for Dementia Research (BDR) cohort with 297,678 genetic variants genotyped on NeuroChip (a custom, dementia‐specific genotyping platform). The dataset is transposed enabling distributed processing. Figure 1 shows the workflow of a novel one‐hot‐encoding framework for transposed data to encode categorical labels into binary arrays resulting in 893,037 entities while maintaining parallelisation and efficiency. A filter‐based feature selection method identified statistically significant genetic variants, reducing the feature‐set from 297,678 to 2,939. Distributed processing ensured scalability, with target labels broadcasted across partitions. distributed machine learning models employing Logistic Regression and SVM validated via 10‐fold cross‐validation. These models are trained on various *p*‐value thresholds to determine optimal feature sets. To evaluate the performance, we have used metrics like AUROC to tackle model‐overfitting.

**Result:**

Our method significantly outperformed existing PRS models, described in achieved an AUROC score of 0.71. In comparison, our global SVM achieved an AUROC score of 0.94. This enhanced accuracy allows better prediction, enabling early AD detection. Additionally, our approach identified 38 novel genetic variants associated with AD, broadening the AD pathogenesis understanding, offering new research directions.

**Conclusion:**

This study provided an AI‐driven approach for the genetic prediction of AD when PRS fails to account for AD's genetic complexity using advanced machine learning techniques. Our methodology improves feature selection, computational efficiency, and predictive accuracy. Identifying novel genetic markers, outperforming PRS models, and enhancing earlier diagnosis for personalized healthcare. We are planning to extend this study with larger datasets by enhancing the proposed feature engineering methods for more robust predictions.